# Pharmacokinetics of 5-Fluorouracil in Patients Treated With Capecitabine Carrying the c.1236G>A *DPYD* Variant Allele

**DOI:** 10.1200/PO-26-00090

**Published:** 2026-06-08

**Authors:** Niels Heersche, Jonathan E. Knikman, Maarten J. Deenen, Hilde Rosing, Ron H.N. van Schaik, Jos H. Beijnen, Jan H.M. Schellens, Hans Gelderblom, Henk-Jan Guchelaar, Jesse J. Swen, Annemieke Cats, Ron H.J. Mathijssen, Bart A.W. Jacobs

**Affiliations:** ^1^ Department of Medical Oncology, Erasmus MC Cancer Institute, Erasmus University Medical Center, Rotterdam, the Netherlands; ^2^ Department of Clinical Chemistry, Erasmus University Medical Center, Rotterdam, the Netherlands; ^3^ Julius Center for Health Sciences and Primary Care, Department of Global Public Health & Bioethics, University Medical Center Utrecht, Utrecht, the Netherlands; ^4^ Department of Clinical Pharmacy, Catharina Hospital, Eindhoven, the Netherlands; ^5^ Department of Clinical Pharmacy and Toxicology, Leiden University Medical Center, Leiden, the Netherlands; ^6^ Department of Pharmacy & Pharmacology, The Netherlands Cancer Institute, Amsterdam, the Netherlands; ^7^ Department of Pharmaceutical Sciences, Utrecht University, Utrecht, the Netherlands; ^8^ Department of Medical Oncology, Leiden University Medical Center, Leiden, the Netherlands; ^9^ Division of Medical Oncology, Department of Gastrointestinal Oncology, The Netherlands Cancer Institute, Amsterdam, the Netherlands

## Abstract

**PURPOSE:**

*DPYD*-guided fluoropyrimidine dosing effectively limits the risk of severe toxicity while maintaining clinical efficacy. However, recent data suggest that c.1236G>A variant carriers, starting with a 25% reduced dose, have shorter progression-free survival than wild-type patients receiving a full dose. Although overall survival was unaffected, further investigation is warranted. To address this, we retrospectively compared 5-fluorouracil (5-FU) exposure between c.1236G>A variant carriers and *DPYD* wild-type patients.

**MATERIALS AND METHODS:**

Pharmacokinetic data from nine clinical trials involving capecitabine-treated patients were pooled. Blood samples were collected before and after administration of capecitabine to assess systemic levels of its metabolites, including 5-FU. Capecitabine dosages were reduced for c.1236G>A variant carriers in accordance with the clinical guidelines at the time of study execution and varied from no reduction (n = 11) to a 25% (n = 16) or 50% (n = 8) reduction. Pharmacokinetic exposure, expressed as area under the plasma concentration-time curve (AUC_0-∞_), was determined using noncompartmental analysis and dose-normalized to 850 mg/m^2^.

**RESULTS:**

In total, 35 heterozygous c.1236G>A patients and 66 *DPYD* wild-type patients were evaluable. Patients carrying c.1236G>A who received a 50% dose reduction had a lower dose-normalized geometric mean 5-FU exposure (234 ng·h/mL coefficient of variation [CV = 43%]) compared with fully dosed c.1236G>A carriers (553 ng·h/mL [CV = 51%]) and fully dosed *DPYD* wild-type patients (582 ng·h/mL [CV = 48%]; *P* < .001). All c.1236G>A carriers who received a 50% dose reduction had AUC_0-∞_ values below the AUC_0-∞_ range observed in the wild-type group.

**CONCLUSION:**

Our findings indicate that an upfront 25% dose reduction for capecitabine in c.1236G>A carriers is likely more appropriate than the currently recommended 50% dose reduction. We stress the importance of individual dose titration in c.1236G>A carriers to avoid both over- and undertreatment.

## INTRODUCTION

**FIG 1.**
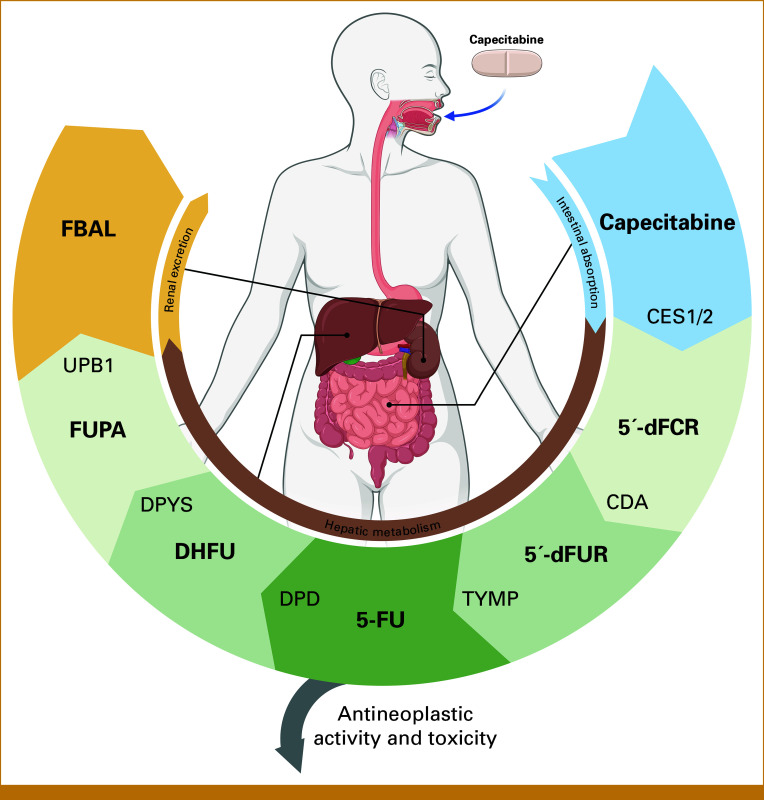
Pharmacokinetics of capecitabine. After oral ingestion, capecitabine is rapidly absorbed in the intestines and then successively metabolized by liver enzymes into 5′-dFCR, 5′-dFUR, 5-FU, DHFU, FUPA, and FBAL. Finally, FBAL is excreted renally. 5-FU is the bioactive metabolite responsible for the drug's antineoplastic effect within tumor cells, but also contributes to toxicity in healthy tissues. Metabolites are shown in bold, and the enzymes involved in each step of the pathway are shown as well. CDA, cytidine deaminase; CES1/2, carboxylesterase 1 and 2; DPD, dihydropyrimidine dehydrogenase; DPYS, dihydropyrimidinase; 5′-dFCR, 5′-deoxy-5-fluorocytidine; 5′-dFUR, 5′-deoxy-5-fluorouridine; 5-FU, 5-fluorouracil; DHFU, 5,6-dihydro-fluorouracil; FBAL, α-fluoro-β-alanine; FUPA, α-fluoro-β-ureidopropionic acid; TYMP, thymidine phosphorylase; UPB1, β-ureidopropionase 1.

Capecitabine, a fluoropyrimidine and oral prodrug of 5-fluorouracil (5-FU), is widely used in the treatment of breast and gastrointestinal tract cancers.^1^ Following ingestion and absorption, capecitabine undergoes extensive enzymatic metabolism, both before and after the formation of the bioactive 5-FU, as shown in Figure 1.^2,3^ The rate-limiting step in the degradation of capecitabine into inactive metabolites is the conversion of 5-FU into 5,6-dihydro-fluorouracil (DHFU) by dihydropyrimidine dehydrogenase (DPD), an enzyme that is encoded by the *DPYD* gene.^1,4-6^

**CONTEXT**

**Key Objective**
To evaluate the adequacy of the current dosing advice, we analyzed pooled pharmacokinetic data on the exposure of capecitabine and its metabolites, including 5-fluorouracil, in individuals carrying the c.1236G>A variant and compared this with wild-type *DPYD* patients.
**Knowledge Generated**
Our findings affirm that patients with the c.1236G>A variant are undertreated when receiving a 50% dose reduction, solidifying the evidence that such a dose reduction may not be suitable for this specific population. Our data underline the importance of preventing undertreatment by performing dose titration based on tolerability.
**Relevance**
On the basis of our pharmacokinetic data, an initial 25% capecitabine dose reduction is more appropriate for patients carrying the c.1236G>A *DPYD* variant rather than the currently recommended 50% dose reduction. Moreover, close monitoring of these patients in subsequent cycles is essential to guide dose modification based on individual tolerability.

Clinically relevant genetic variants in *DPYD*—present in 3%-8% of all patients, depending on the studied population—impair DPD enzyme functionality and significantly reduce 5-FU metabolism.^4,7,8^ Consequently, DPD-deficient patients are prone to experience severe fluoropyrimidine-related gastrointestinal and hematologic toxicities due to 5-FU overexposure. Moreover, these patients have a markedly increased risk of fatal toxicity (odds ratio: 34.9; 95% confidence interval (95% CI, 14.0 to 87.1; *P* < .05).^9^ The most commonly tested and clinically validated *DPYD* variants include c.1679T>G (rs55886062, DPYD*13, I560S), c.2846A>T (rs67376798, D949V), c.1905+1G>A (rs3918290, DPYD*2A, IVS14+1G>A), and c.1236G>A (rs56038477, E412E).^4,7,10^ The c.1236G>A variant is in near-complete linkage disequilibrium with the intronic variant c.1129-5923C>G (rs75017182), which has been identified as the causal variant responsible for reduced DPD activity.^11-14^ Jointly, these two variants form haplotype B3.^11-14^ Pretherapeutic genotyping for these common variants in *DPYD*, followed by a dose reduction in variant carriers, has been shown to mitigate toxicity.^15,16^ Accordingly, the European Medicines Agency and US Food and Drug Administration recommend testing for DPD deficiency before initiation of fluoropyrimidine treatment.^17,18^

Despite these recommendations, concerns persist regarding the impact of performing dose reductions in *DPYD* variant carriers on survival outcomes, including progression-free survival (PFS) and overall survival. In 2023, our research group conducted a retrospective matched-pair analysis in 93 *DPYD* variant carriers and found that *DPYD-*guided fluoropyrimidine dosing did not affect progression-free and overall survival. However, an explorative subgroup analysis of 61 c.1236G>A variant carriers, who started with a 25% dose reduction, suggested a shorter PFS compared with wild-type patients receiving a full dose (hazard ratio, 1.43 [95% CI, 1.10 to 1.86]; *P* = .007).^19^ Although the overall survival of the c.1236G>A carriers was unaffected, this unexpected finding raised concern about the appropriateness of a 25% dose reduction for these patients. This is particularly important given that the current guidelines advise an even larger dose reduction of 50% for c.1236G>A carriers,^4,20^ as a 25% dose reduction was insufficient to normalize rates of severe toxicity in these patients.^16^ These conflicting findings are cause of ongoing debate regarding the optimal dosage for c.1236G>A carriers and warrant further evaluation.^21,22^ Hence, we conducted an analysis of pooled pharmacokinetic data to assess the systemic exposure of 5-FU in patients treated with capecitabine carrying the c.1236G>A variant.

## MATERIALS AND METHODS

### Design

Pharmacokinetic data were pooled from nine previously conducted prospective studies that measured plasma concentrations of capecitabine and its metabolites in patients with cancer. Studies were selected based on predefined inclusion and exclusion criteria, and patient eligibility was assessed accordingly. This retrospective pooled analysis compared exposure to capecitabine and its metabolites in carriers of the c.1236G>A variant with exposure in *DPYD* wild-type patients (ie, absence of DPYD*13, c.2846A>T, DPYD*2A, and c.1236G>A). All studies were conducted either in full or in part at the Netherlands Cancer Institute and/or the Erasmus Medical Center Cancer Institute. Each study was approved by the Medical Ethics Committee of either institute and complied with the principles laid out in the Declaration of Helsinki.^23^

### Study Population

The study population was retrieved from nine previously conducted trials in which pharmacokinetic sampling was performed in capecitabine-treated patients, some of which also included patients heterozygous for c.1236G>A.^16,24-32^ Patients carrying a c.1236G>A variant were pooled to form the c.1236G>A carrier cohort.^16,24-30^ Additionally, wild-type *DPYD* patients for whom rich pharmacokinetic data were available were pooled in the *DPYD* wild-type cohort.^30-32^ A global overview of the characteristics of these studies is presented in the Data Supplement (Table S1).

Patients were considered eligible for evaluation if they were genotyped for the four commonly tested *DPYD* variants, being DPYD*13, c.2846A>T, DPYD*2A, and c.1236G>A. Genotyping was performed either before capecitabine treatment^16,29,31,32^ or retrospectively as part of observational research into *DPYD*.^24-28,30^ Patients who were genotyped reactively due to the onset of severe fluoropyrimidine-related toxicity were excluded from the analysis to prevent selection bias. *DPYD* wild-type status was defined as the absence of all four tested *DPYD* variants. Moreover, body surface area (BSA) of the patient and their respective dose of capecitabine had to be available. In addition to 5-FU, quantified concentrations of capecitabine, 5′-deoxy-5-fluorocytidine (5′-dFCR), 5′-deoxy-5-fluorouridine (5′-dFUR), and α-fluoro-β-alanine (FBAL) were accrued for exploratory analysis, if available.

### Analysis

Concentrations of capecitabine, 5′-dFCR, 5′-dFUR, 5-FU, and FBAL were quantified using four validated and previously reported liquid chromatography-tandem mass spectrometry (LC-MS/MS) assays (Data Supplement, Table S2).^32-35^ A noncompartmental pharmacokinetic analysis was used to calculate individual exposure to each of the metabolites, expressed as area under the plasma concentration-time curve (AUC) in ng·h/mL. The primary objective of the study was to determine the difference in AUC_0-∞_ of 5-FU between the c.1236G>A cohort and the *DPYD* wild-type cohort. Secondary objectives were to determine the differences in AUC_0-∞_ of the other metabolites. AUC_0-∞_ values were dose-normalized and log-transformed for analysis, assuming pharmacokinetic exposure to follow a log-normal distribution.^36,37^ Data were analyzed using an unpaired t-test, and relative differences with accompanying 95% CI were calculated. Sensitivity analyses were conducted to assess the impact of different assays, sampling schedules, and sampling days used in the included studies by comparing exposure levels across studies. As a subgroup analysis, dose-normalized AUC_0-∞_ values were compared across dose reduction subgroups within the c.1236G>A cohort (ie, no dose reduction, 25% reduction, or 50% reduction of the capecitabine dose). Baseline demographic characteristics were collected and compared between both cohorts using either a Chi-square or Mann-Whitney U test. Statistical tests were performed using R statistical software (version 4.2.1). *P* values <.05 were considered significant. Complete details relating to the bioanalytical assays, the NCA, and statistics are available in the Data Supplement, Methods.

## RESULTS

A total of 36 heterozygous c.1236G>A carriers were identified from seven clinical trials. Of note, 12 c.1236G>A carriers were treated at a full dose, whereas 16 and 18 c.1236G>A carriers were treated with a 25% and 50% dose reduction, respectively. Additionally, 68 *DPYD* wild-type patients with available pharmacokinetic data were included from three clinical trials^30-32^ to form the *DPYD* wild-type cohort. All 104 patients met the inclusion criteria, although one patient in the c.1236G>A carrier cohort and two patients in the *DPYD* wild-type cohort were excluded from the analysis as their AUC for 5-FU did not meet the criteria for extrapolation to infinity.

The majority of evaluable patients was male (58%) and of European ancestry (95%). Although tumor type and treatment setting varied widely, the predominant malignancies were colorectal, gastric, and esophageal cancers with most patients having metastatic disease. A small subset of patients (8%) underwent upper gastrointestinal surgery for their tumor before capecitabine treatment (ie, esophagectomy, or partial or total gastrectomy). Median dose intensity for a single dose of capecitabine was 681 mg/m^2^ (IQR, 548-776 mg/m^2^) for c.1236G>A carriers, compared with 778 mg/m^2^ (IQR, 728-852 mg/m^2^) in the *DPYD* wild-type cohort. Full baseline characteristics are described in Table 1.

**TABLE 1.** Baseline Characteristics of Evaluable Patients

Characteristic	c.1236G>A Carrier Cohort (n = 35)		*DPYD* Wild-Type Cohort (n = 66)		*P*
	Number (n)	Percentage (%)	Number (n)	Percentage (%)	
Sex					.30
Female	17	49	25	38	
Male	18	51	41	62	
Age (years), median (IQR)	60 (51-70)		59 (53-66)		.59
Ethnicity					>.99
White	33	94	63	96	
Other^a^	2	6	3	4	
Body surface area (kg/m^2^), median (IQR)	1.96 (1.71-2.04)		1.98 (1.80-2.08)		.44
WHO performance status					.18
0	19	54	24	36	
1	15	43	41	62	
2	1	3	1	2	
Primary tumor					<.01
Colorectal	17	48	24	36	
Gastric	8	23	10	15	
Esophagogastric junction	3	9	17	26	
Other^b^	7	20	15	23	
Tumor stage					<.01
Local	3	9	—	—	
Locally advanced	12	34	5	8	
Metastatic	20	57	61	92	
Prior upper gastrointestinal surgery^c^					.86
No	32	91	61	92	
Yes	3	9	5	8	
Capecitabine dose (mg), median (range)	1,300 (1,000-1,500)		1,500 (1,450-1,650)		NA
*DPYD* genotype					NA
*1/*1	—	—	66	100	
*1/c.1236G>A	35	100	—	—	

Abbreviations: *DPYD*, dihydropyrimidine dehydrogenase gene; kg/m^2^, kilogram per square meter; mg, milligram; n, number; NA, not applicable.

^a^ In the c.1236G>A carrier cohort, one patient of Surinamese and one of Asian descent were included. Within the *DPYD*, wild-type cohort, four patients of Surinamese, Turkish, Asian, and Black descent were included.

^b^ Other primary tumors in the c.1236G>A carrier cohort were anal (n = 3), breast (n = 2), esophageal cancer (n = 1), and mucinous adenocarcinoma (n = 1); for the *DPYD*, wild-type cohort, these included head and neck (n = 4), esophageal (n = 2), neuroendocrine carcinoma (n = 2), cancer of unknown primary (n = 1), thyroid (n = 1), breast (n = 1), ovarian (n = 1), gall bladder (n = 1), small cell lung cancer (n = 1), and pancreatic cancer (n = 1).

^c^ That is, esophagectomy or partial or total gastrectomy.

### Evaluability of Pharmacokinetic Data

Individual AUC_0-∞_ for capecitabine and 5-FU was available in 101 of 104 patients (97%). Exposure to 5′-dFCR and 5′-dFUR could be extrapolated in 77 and 76 of 79 curves (97% and 96%), respectively. For FBAL however, AUC_0-∞_ was obtained in only 50 of 79 curves (63%). Hence, FBAL was excluded from the final analysis. The most common reason for invalid FBAL AUC_0-∞_ was exceedance of the pre-established +20% extrapolation limit. Sensitivity analyses grouping patients by assay method, sampling schedule, or sampling day showed no significant impact of these factors on individual exposure.

### Exposure to 5-FU

**FIG 2.**
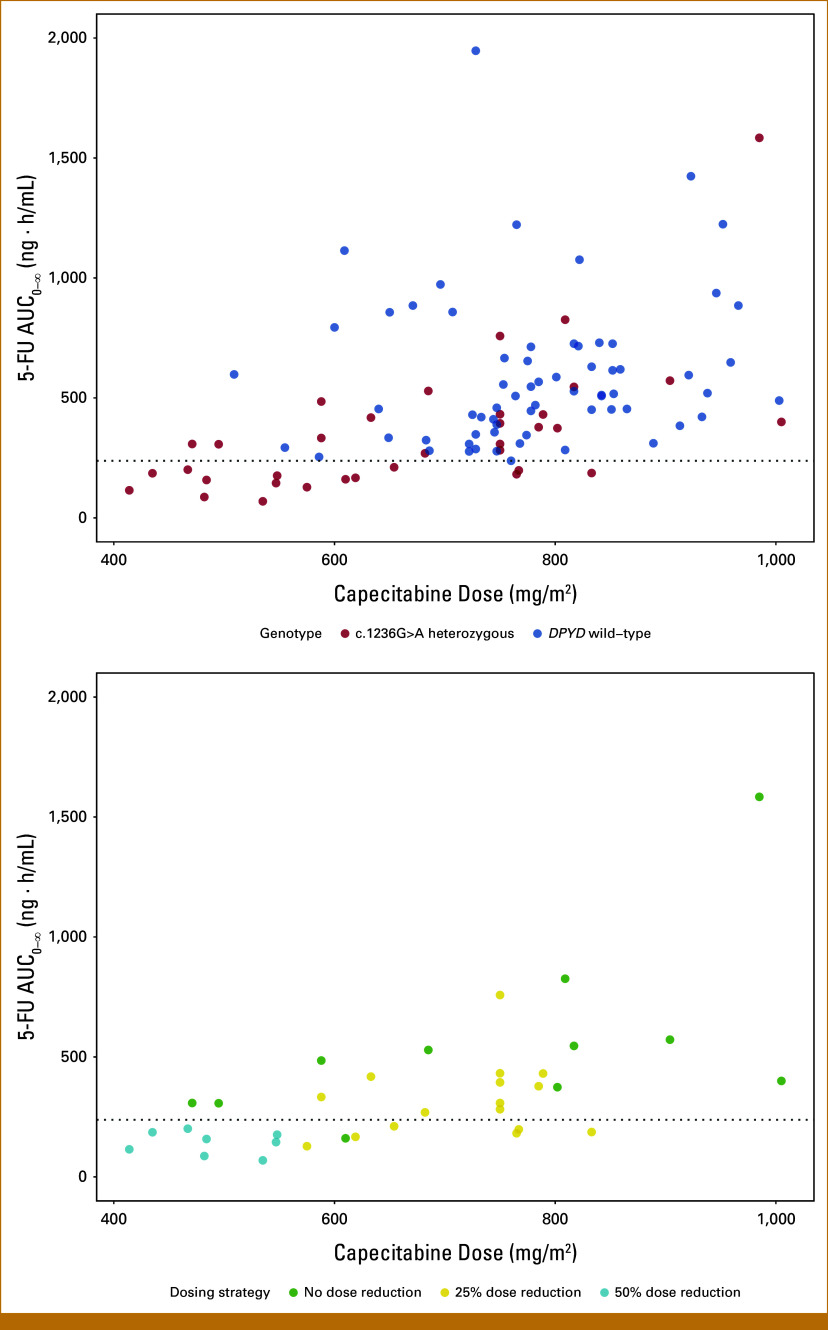
Individual exposure to 5-FU. Exposure to 5-FU is plotted against the capecitabine dose in mg/m^2^. This is presented for the total cohort (upper panel), stratified by genotype (ie, c.1236G>A [red] *v DPYD* wild-type [blue]) and for c.1236G>A carriers (lower panel), stratified by dosing strategy (ie, no dose reduction [green], or a 25% [yellow] or 50% dose reduction [light blue], respectively); gray dotted line indicates the lower limit of the AUC_0-∞_ range as seen in *DPYD* wild-type patients (ie, 238 ng·h/mL after a capecitabine dose of 760 mg/m^2^). AUC_0-∞_, area under the plasma concentration-time curve, from zero to infinity, expressed in nanogram times hour per milliliter; *DPYD*, dihydropyrimidine dehydrogenase gene; 5-FU, 5-fluorouracil; mg/m^2^, milligram per square meter.

**FIG 3.**
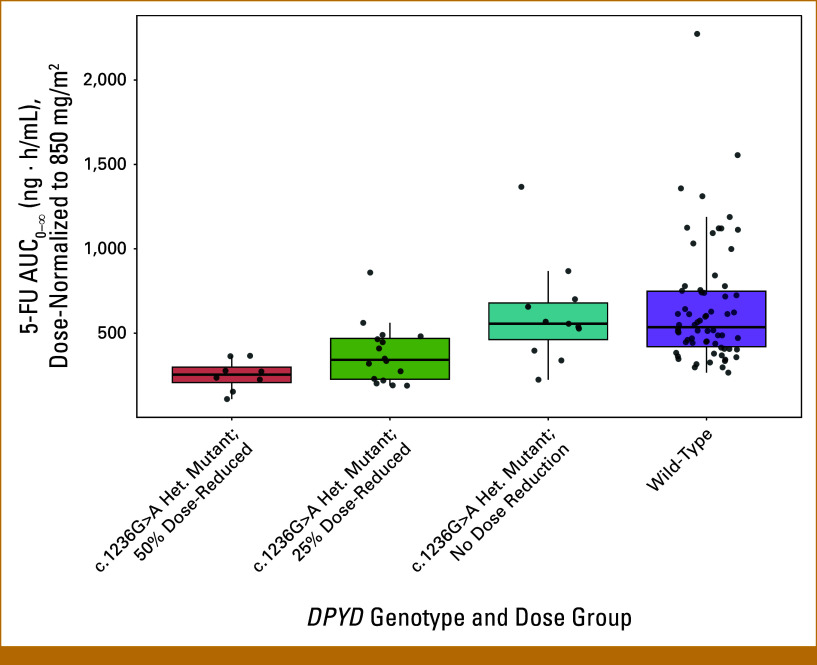
Exposure to 5-FU across *DPYD* and dose subgroups Box plots show median and interquartile range for individual dose-corrected AUC_0-∞_ for c.1236G>A carriers, grouped by administered dose (50%, 75%, or 100% of standard dose), as well as for *DPYD* wild-type patients. AUC_0-∞_, area under the plasma concentration-time curve, from zero to infinity, expressed in nanogram times hour per milliliter; *DPYD*, dihydropyrimidine dehydrogenase gene; 5-FU, 5-fluorouracil; het., heterozygous; mg/m^2^, milligram per square meter.

Dose-normalized exposure to 5-FU was significantly lower among c.1236G>A carriers compared with *DPYD* wild-type patients. Whereas wild-type patients had a geometric mean AUC_0-∞_ of 5-FU of 582 ng·h/mL (coefficient of variation (CV) = 48%), c.1236G>A carriers exhibited an AUC_0-∞_ of 5-FU of 365 ng·h/mL (CV = 58%). This constituted a statistically significant lower exposure of –37% (95% CI, –49% to –23%; *P* < .001). Individual, absolute 5-FU exposures are presented in Figure 2A. Notably, all c.1236G>A patients who received a 50% dose reduction had exposure values below the lower limit of the AUC_0-∞_ range observed in the wild-type group (see Fig 2B). Subgroup analysis revealed significant differences among fully dosed, 25% dose-reduced, and 50% dose-reduced c.1236G>A carriers, with geometric mean AUC_0_-∞ values of 553 ng·h/mL (CV = 51%), 343 ng·h/mL (CV = 46%), and 234 ng·h/mL (CV = 43%), respectively (ANOVA *P* < .001). Tukey post hoc analysis confirmed significantly lower 5-FU exposure in the 25% (*P* < .001) and 50% (*P* < .001) dose-reduced subgroups compared with fully dosed c.1236G>A heterozygous carriers and wild-type patients (Fig 3).

Exposure to capecitabine, 5′-dFCR, and 5′-dFUR is presented in Table 2, and complete results including exposures across dose-reduction subgroups in the c.1236G>A carrier cohort are available in the Data Supplement, Results and Figures S1-S3.

**TABLE 2.** Exposure to Capecitabine and Its Metabolites, Values Are Dose-Corrected to 850 mg/m^2^

Metabolite	Geometric Mean AUC_0-∞_ (ng·h/mL) (CV%)									
	*DPYD* Wild-Type Cohort		c.1236G>A Heterozygous Carrier Cohort							
			All c.1236G>A Carriers		Subgroups of c.1236G>A Heterozygous Carrier Cohort					
		n		n	50% Dose Reduction	n	25% Dose Reduction	n	No Dose Reduction	n
Capecitabine	4,443 (46%)	66/68	5,602 (72%)	35/36	6,021 (64%)	8/8	5,481 (88%)	16/16	5,486 (60%)	11/12
5′-dFCR	9,176 (35%)	42/43	6,888 (133%)	35/36	3,784 (596%)	8/8	9,234 (50%)	16/16	6,951 (45%)	11/12
5′-dFUR	8,223 (23%)	42/43	9,491 (36%)	34/36	9,625 (35%)	8/8	9,663 (37%)	16/16	9,120 (40%)	10/12
5-FU	582 (48%)	66/68	365 (58%)	35/36	234 (43%)	8/8	343 (46%)	16/16	553 (51%)	11/12
FBAL	NA	29/43	NA	21/36	NA	6/8	NA	10/16	NA	5/12

Abbreviations: AUC_0-∞_, area under the plasma concentration-time curve, from zero to infinity; CV, coefficient of variation; 5′-dFCR, 5′-deoxy-5-fluorocytidine; 5′-dFUR, 5′-deoxy-5-fluorouridine; 5-FU, 5-fluorouracil; *DPYD*, dihydropyrimidine dehydrogenase gene; FBAL, α-fluoro-β-alanine; n, number of evaluable patients; NA, not available; ng·h/mL, nanogram times hour per milliliter.

## DISCUSSION

To our knowledge, this is the first study to specifically analyze the pharmacokinetics of capecitabine in patients who harbor the most common, clinically relevant *DPYD* variant, that is, the c.1236G>A variant. As far as we are aware, the current study also represents the largest cohort of c.1236G>A carriers to describe fluoropyrimidine pharmacokinetics to date. Our analysis found a lower dose-normalized exposure to 5-FU in c.1236G>A carriers compared with wild-type patients. Interestingly, subgroup analysis showed that among 11 c.1236G>A carriers receiving a full capecitabine dose, the exposure to 5-FU was comparable to that of wild-type patients (geometric means: 553 *v* 582 ng·h/mL, respectively). Contrarily, c.1236G>A carriers receiving a 50% dose reduction of capecitabine had the lowest exposures, with all AUC values below the range seen in wild types. These findings suggest that the current dosing guidelines^4,20^ for fluoropyrimidines in patients with the c.1236G>A variant—recommending an initial 50% dose reduction followed by up-titration—result in underdosing in a substantial subset of patients in the initial cycles, which could compromise treatment efficacy. This is especially true when the capecitabine dose (in discordance with the current guidelines) is not ramped up in the absence of relevant toxicity. This finding was unexpected, as upfront dose reductions are intended to normalize pharmacokinetic exposure by compensating for reduced 5-FU metabolism in *DPYD* variant carriers.^38^ Notably, among the four most commonly tested *DPYD* variants, c.1236G>A is the most frequent, accounting for approximately 60% of genotype-derived partial DPD deficiency cases in Western populations, emphasizing the importance of determining the right starting dose for these patients.^4,8,16^

Pharmacokinetic data in patients with the c.1236G>A variant are sparsely reported in literature. In a preliminary analysis of a small set of c.1236G>A carriers, we previously did not find evidence for an altered 5-FU clearance following capecitabine administration compared with wild-type patients.^39^ This aligns with our current finding that carriers of the c.1236G>A variant receiving dose reductions show lower AUCs compared with fully dosed wild-type patients. In our analysis, we also identified a counterintuitive 37% lower dose-normalized geometric mean 5-FU AUC in c.1236G>A carriers compared with fully dosed wild-type patients. However, in fully dosed c.1236G>A mutation carriers, no reduced 5-FU exposures were found, indicating that the 37% lower AUC is likely the result of the performed dose-normalization. It seems that dose reductions in variant carriers may disproportionately affect 5-FU exposure. Our dose-correction method assumed a linear relationship—that is, that a 50% dose reduction would result in a 50% lower AUC. In practice however, the reduction in exposure appears to be greater, indicating that our correction method underestimated AUC values of dose-reduced c.1236G>A carriers after dose-normalization. Early phase I trials of capecitabine in patients with unknown *DPYD* status support this, as in those trials dose reductions of capecitabine with 25% and 50% led to decreases in exposure to 5-FU with 40% and 75%, respectively.^3,40-42^ In conclusion, our data show that dose reductions with 50% for c.1236G>A carriers result in underexposure in a substantial number of patients, whereas at a full capecitabine dose, exposure in c.1236G>A carriers to 5-FU is comparable to wild-type patients receiving a full dose.

Interestingly, recent data by Fiebrich-Westra et al^43^ seem to confirm the aforementioned finding. In their study, 16 heterozygous carriers of c.1236G>A received a 50% reduced dose of intravenous 5-FU and were subsequently subjected to therapeutic drug monitoring. Among their patients, all but one were below the therapeutic threshold of 5-FU at the initial 50% dose. To achieve an AUC within the target range in c.1236G>A carriers, a median dose of 75% of the standard 5-FU dose was required. They hypothesized that at lower doses, 5-FU metabolism is not saturated, regardless of the c.1236G>A mutation.

Several studies did report DPD enzyme activity of patients with a c.1236G>A variant.^10,13,16,44^ Although these studies consistently report a mean decrease of about 20%-25% in enzyme activity, they also highlight a substantial interindividual variability among c.1236G>A carriers, with DPD activity ranging from normal levels to as much as a 50% reduction.^10,13,16,44^

The initial dosing advice for c.1236G>A carriers was to perform a 25% dose reduction on the basis that c.1236G>A carriers have a roughly 20%-25% lower DPD enzyme activity compared with wild-type patients.^10^ However, in the first trial to prospectively study *DPYD-*guided dosing for fluoropyrimidines in c.1236G>A carriers, a 25% dose reduction was insufficient to statistically normalize the risk of severe fluoropyrimidine-related toxicity (relative risk for severe toxicity after 25% dose reduction: 1.69 [95% CI, 1.18 to 2.42]).^16^ Hence, guidelines were revised to recommend a larger 50% dose reduction, followed by incremental dose escalation in case treatment is well tolerated.^4,20^ These findings, along with the current analysis, underscore the narrow therapeutic window for fluoropyrimidines in c.1236G>A variant carriers, emphasizing the need for precise individualized dosing. Consequently, physicians must carefully balance the risk of suboptimal treatment due to underdosing against the risk of severe and potentially life-threatening toxicity due to excessive dosing. This is particularly relevant for short-course fluoropyrimidine regimens, such as adjuvant therapy or chemoradiotherapy, where limited treatment duration restricts optimal dose titration.

In our view, the optimal initial dose of capecitabine for patients carrying the c.1236G>A variant remains unclear due to the conflicting evidence regarding both safety and survival. Nonetheless, the present pharmacokinetic analysis of capecitabine suggests that a 25% initial dose reduction may be more appropriate than the currently recommended 50% reduction. We further emphasize the importance of subsequent dose titration after a pre-emptive reduction in patients to prevent over- and undertreatment. Finally, it is important to note that systemic 5-FU concentrations do not necessarily represent intratumoral 5-FU concentrations as capecitabine is preferentially activated into 5-FU by tumor cells, and that clear exposure-response and exposure-toxicity relationships are lacking in capecitabine treatment.^3,45,46^ Therefore future studies should aim to find the optimal capecitabine dosage for c.1236G>A carriers based on clinical outcomes.

Our analysis found a significantly 15% higher 5′-dFUR exposure in patients harboring the c.1236G>A variant. The statistical significance of this finding is likely a reflection of the high interpatient variation and should not be considered clinically relevant. Additionally, we were unable to adequately extrapolate FBAL exposure due to its comparatively late time to peak plasma concentration (3.34 hours) and extended half-life (3.23 hours), especially in relation to the employed sampling schedules.^1^ However, as FBAL is not a clinically active metabolite of capecitabine, its exclusion from our analysis is unlikely to affect interpretation of our data.^1^ For the other metabolites, this limitation was not present and extrapolation to infinity was rarely required. A small number of profiles were excluded due to atypical absorption kinetics, in which the elimination phase had not commenced by the end of sampling. Although population pharmacokinetic modeling could have addressed these challenges, the use of NCA allowed us to ensure that our results remain readily comparable with previously published clinical studies, where similar methodologies were applied. Moreover, we captured a wide range of capecitabine dosages by incorporating phase I dose-finding studies, enabling the exploration of capecitabine pharmacokinetics across multiple dosing levels in wild-type patients. Additionally, some studies were performed before the implementation of *DPYD*-guided dosing of fluoropyrimidines, meaning c.1236G>A carriers received a full capecitabine dose, whereas more recent studies used a 25% or 50% dose reduction for patients carrying c.1236G>A. This enabled assessment of the differences in exposure across the full dosing range for c.1236G>A carriers. The current analysis also has several limitations. Although this is the largest pharmacokinetic analysis of c.1236G>A carriers to date, the total number of patients remains relatively limited. Additionally, the retrospective nature of our analysis led to heterogeneity in used assays, sampling schedules, and day of sampling. However, all assays were (cross-)validated to ensure accurate quantification. Additionally, sensitivity analyses found no differences in exposure between the different assays, sampling schedules, or day of sampling.

Eight patients (8%) underwent (partial) gastrectomy or esophagectomy before capecitabine treatment, which may result in more rapid absorption of capecitabine and increased exposure to 5-FU.^39^ Indeed, the one patient who underwent total gastrectomy had a relatively high AUC_0-∞_ of 5-FU of 1,076 ng·h/mL after a capecitabine dose of 822 mg/m^2^. For the other patients who underwent esophagectomy or partial gastrectomy, this effect was less pronounced. Since the incidence of prior upper gastrointestinal surgery was comparable among both genotype groups (8% *v* 9%; *P* = .86), we believe this did not affect our conclusions.

In summary, this study investigated the pharmacokinetics of capecitabine and its metabolites in a cohort of c.1236G>A carriers and compared it with *DPYD* wild-type patients. Our findings demonstrate that the currently recommended 50% dose reduction for c.1236G>A heterozygous carriers results in clinically relevant underexposure to 5-FU. This indicates that based on these pharmacokinetic outcomes, an initial 25% dose reduction is likely more appropriate for carriers of the c.1236G>A variant treated with capecitabine than the currently advised 50% dose reduction. We also recommend individualized dose titration based on tolerability in subsequent cycles.

## Data Availability

The data that support the findings of this study are available from the corresponding author upon reasonable request.
